# MDM2: current research status and prospects of tumor treatment

**DOI:** 10.1186/s12935-024-03356-8

**Published:** 2024-05-13

**Authors:** Yumei Yao, Qian Zhang, Zhi Li, Hushan Zhang

**Affiliations:** 1 Zhaotong Health Vocational College, No 603 Yucai Road, Zhaotong City, Yunnan Province 657000 People’s Republic of China; 2 Anning First People’s Hospital Affiliated to Kunming University of Science and Technology, Kunming, Yunnan 650302 People’s Republic of China

## Abstract

Mousedouble minute 2 (MDM2) is one of the molecules activated by p53 and plays an important role in the regulation of p53. MDM2 is generally believed to function as a negative regulator of p53 by facilitating its ubiquitination and subsequent degradation. Consequently, blocked p53 activity often fails in damaged cells to undergo cell cycle arrest or apoptosis. Given that around 50% of human cancers involve the inactivation of p53 through genetic mutations, and directly targeting p53 through drug development has limited feasibility, targeting molecular regulation related to p53 has great potential and has become a research hotspot. For example, developing drugs that target the interaction between p53 and MDM2. Such drugs aim to reactivate p53 by targeting either MDM2 binding or p53 phosphorylation. Researchers have identified various compounds that can serve as inhibitors, either by directly binding to MDM2 or by modifying p53 through phosphorylation. Furthermore, a significant correlation exists between the expression of MDM2 in tumors and the effectiveness of immunotherapy, predominantly in the context of immune checkpoint inhibition. This review presents a comprehensive overview of the molecular characteristics of MDM2 and the current state of research on MDM2-targeting inhibitors. It includes a review of the impact of MDM2 targeting on the efficacy of immunotherapy, providing guidance and direction for the development of drugs targeting the p53-MDM2 interaction and optimization of immunotherapy.

## Introduction of MDM2 and p53

The transcription factor p53 plays a crucial role in cell cycle progression, apoptosis, senescence, DNA repair and metabolism, and has a powerful tumor-suppressive function. Studies have shown that mice lacking p53 protein develop normally but are prone to various types of tumors [1]. *TP53*, the gene encoding p53, is mutated or deleted in around 50% of human cancers, impairing p53’s function to suppress tumor [2]. Since p53 plays a key role in regulating numerous cellular processes, its levels and activity are intricately controlled, with MDM2 serving as one of its main cellular modulators. MDM2 is a regulatory protein comprising 491 amino acids, consisting of an N-terminal p53 binding domain, a nuclear localization signal (NLS), a nuclear export signal (NES), an acidic structural domain, a zinc finger structural domain, and a C-terminal RING-finger domain. The NLS and NES are crucial for MDM2's transport from the nucleus to the cytoplasm. Within the acidic region, residues are phosphorylated to induce the degradation of p53. Additionally, the C-terminal RING finger structural domain functions as an E3 ubiquitin ligase, inducing p53 ubiquitination after p53-MDM2 interaction. Additionally, the C-terminal RING domain, as an E3 ubiquitin ligase, facilitates p53 ubiquitination upon interaction of MDM2-p53 [3]. MDM2 binds to the C-terminal oligomerization structural domain of p53, inducing its ubiquitination and subsequent proteasomal degradation, thereby regulating the cell cycle. Mutations that disrupt or impair the function of the p53 oligomerization domain result in decreased binding affinity of MDM2 to p53, affecting the proteasomal degradation of p53 [4]. On the other hand, the zinc finger structural domain adjacent to the acidic structural domain of MDM2 regulates p53 levels by interacting with ribosomal proteins that bind to the acidic structural domain, thereby inhibiting p53 degradation [5]. The MDM2 gene expressing this protein consists of 12 exons, including two promoters of p53 response element (RE), P1 and P2, which regulate the expression of two MDM2 proteins, p90 and p76, respectively. P90 is a full-length and functional protein that can bind to p53 in the p53 binding domain. In contrast, p76 is shorter than p90 and acts as a negative inhibitor of p90 to activate p53 due to its lack of a p53-binding domain [6]. Next to the two promoters, there are ten additional exons, the first two of which are involved in the translation of p90 and p76, using ATG as the first start codon. P90 and p76 are translated from exons 3 and 4, respectively. P76-MDM2 inhibits the ability of p90-MDM2 to destabilize p53 stability.

Many small molecules induce post-translational modifications of p53, including phosphorylation, acetylation, ubiquitination, sumoylation and methylation, thereby preventing its degradation and increasing stability and transcriptional activity [7–12]. DNA-damaging agents, such as ultraviolet (UV) radiation, ionizing radiation, and cisplatin compounds, induce p53 phosphorylation-dependent stabilization [13]. Among the several phosphorylation sites in p53, Ser15, Thr18, and Ser20 play a key role in the interaction with MDM2 [14]. DNA-dependent p53 modification acetylation promotes chromatin remodeling and activates the expression of p53 target genes [15]. The acetylated residues of p53 are mainly located at its C-terminal, among which the most important ones are K370, K372, K373, K381, K382, and K386, which are acetylated by the highly related histone acetyltransferase p300 and cAMP reaction element (CREB)-binding protein (CBP) [16]. These lysine residues are targeted by MDM2 for ubiquitination [17]. In addition to its ubiquitination activity, MDM2 also promotes neural precursor cell expressed developmentally downregulated protein 8 (Nedd8). Nedd8 is another ubiquitin-like enzyme that can be coupled to the lysine residue of p53 by the sumoylation process [18–20], and exerts its inhibitory function by binding to p53.

As mentioned above, the MDM2 gene acts as a ubiquitin ligase and is a negative regulator of p53 [21, 22]. It first physically interacts with p53 at the transactivation domain (TAD), which is essentially amphipathic. The binding pocket within the N-terminal p53 binding domain of MDM2 is essentially hydrophobic [23]. The p53 amino acid residues, including Phe19, Trp23, and Leu26, form the hydrophobic side of the TAD amphipathic α-helix. Although Phe19, Trp23, and Leu26 are not the only residues that form α-helices in p53, they play a key role in the interaction between p53 and MDM2. Once the hydrophobic α-helix is formed, it contacts the hydrophobic binding pocket of MDM2, forming the p53/MDM2 complex through hydrogen bonds. In this interaction, MDM2 induces the ubiquitination of p53 at the N-terminal transactivation domain, leading to proteasomal degradation and inhibition of its transcriptional activity.

One of the target genes of p53 is the cyclin-dependent kinase (CDK) inhibitor p21, which is a negative regulator of the cell cycle [24]. If p53 is mutated, p21 will not be activated, causing DNA damage and leading to cell cycle progression [25]. As mentioned earlier, many cancers inactivate p53, leading to the progression of the cell cycle and the survival of cancer cells. Therefore, the p53-MDM2 interaction is a key focus in cancer treatment research. Current cancer treatment methods aim to restore p53 function by inhibiting the interaction between p53 and MDM2 or by degrading MDM2, to increase p53 levels and prevent the survival and proliferation of tumor cells.

## Correlation between MDM2 and tumorigenesis

In acute myeloid leukemia (AML), P53 pathway dysregulation occurs through various mechanisms [26, 27], with less than a quarter of AML cases being due to changes in the TP53 gene itself, while others involve downregulation through other mechanisms, such as increased MDM2 activity. MDM2 inhibits p53-dependent transcription, promotes its nuclear export, and facilitates its degradation, thereby reducing p53 activity [28]. In more than half of leukemia cases, MDM2 is overexpressed and associated with adverse karyotypes [29]. Therefore, MDM2 is a therapeutic target for TP53 wild-type (WT) AML, and small molecule antagonists of MDM2, called nutlins, have been investigated in clinical trials for AML and myeloproliferative neoplasms (MPN) [30, 31]. Nutlins and similar inhibitors disrupt the MDM2 and p53 interaction by binding to the p53-binding pocket [32]. These compounds have significant clinical activity in AML and MPN, and high expression levels of MDM2 in AML blasts have been shown to correlate with clinical response [33].

MDM2 influences tumor development and progression in breast cancer through multiple pathways. Firstly, P21 is a downstream cell cycle protein kinase inhibitor of P53, and in breast cancer, it can block cell cycle progression, exerting an anti-cancer effect. MDM2 can directly bind to the C8 subunit of 20S proteasome in P21 independently of P53, promote P21 degradation, further promote the progression of breast cancer and reduce its sensitivity to chemotherapy [34]. Secondly, estrogen receptor alpha (ERα) is a nuclear receptor and an oncoprotein expressed in about 70% of breast cancers. The expression of MDM2 is positively correlated with ERα expression in human breast cancer tissues and cell lines. ERα positively regulates MDM2 expression through a P53-independent pathway, and MDM2 promotes the ubiquitination and degradation of ERα by forming a protein complex with it [35]. In addition, the tumor suppressor PTEN inhibits the PI3K/AKT signaling pathway, while AKT promotes transcription of MDM2 gene, and the entry of MDM2 protein into the nucleus and phosphorylation. Therefore, PTEN will lead to the aggregation of MDM2 in the cytoplasm, and PTEN deletion will activate the MDM2-mediated anti-apoptosis process, resulting in abnormal proliferation of breast cancer cells [36]. Furthermore, PTEN can regulate MDM2 promoter region transcription and the selection of homologous isoforms through lipid phosphatases. Epithelial-mesenchymal transition (EMT) is a crucial process in the migration and invasion of malignant tumor cells derived from epithelial tissue. Multiple signaling pathways are involved, and MDM2 upregulates EMT-related transcription factors through the B-Raf signaling pathway or induces EMT by enhancing Snail expression, promoting breast cancer progression [37]. MDM2 acts directly as a carcinogenic agent, MDM2 protein can act as an oncogene when it is overexpressed, or influence disease progression through different MDM2 splicing variants and post-translational modifications. Studies have shown that in 5.9% of breast cancer patients, the MDM2 gene was amplified approximately 17-fold in tumor cells, and there were post-translational modified MDM2 splice variants. In patients with breast cancer who have alternatively spliced MDM2 transcripts, these variants tend to be more biologically aggressive, with greater axillary lymph-node involvement and greater necrosis [38]. MDM2 splice variants MDM2-B and MDM2-C have longer half-lives than full-length MDM2 protein after chemotherapy, and MDM2-C can act as an E3 ubiquitin ligase to induce apoptosis in breast cancer cells [39]. Splice variants P2-MDM2-10 and MDM2-5 are significantly upregulated in stressed cells, suggesting that alternative splicing may influence the oncogenic activity of the MDM2 gene [40].

Changes in the MDM2-p53 pathway are common in primary hepatocellular carcinoma (HCC) [31, 41, 42]. In HCC, researchers have revealed that MDM2 expression and the MDM2-p53 pathway are regulated by various molecular mechanisms [43]. Mutations in TP53 in HCC mainly occur in the DNA-binding domain of p53, leading to the reduced affinity for sequence-specific response elements of its target genes. This reduces p53-mediated MDM2 induction, and MDM2 dysregulation results in increased levels of mutant p53 in tumor cells or tissues [44]. It has been reported that the expression of the tumor suppressor KLF6 is negatively correlated with the prognosis of HCC [45]. KLF6 is a member of the Krüppel-like C2H2 zinc finger family, known to be involved in cell cycle regulation, signal transduction, and cell differentiation. KLF6 may also function as a negative regulator of MDM2. Milco et al. reported that reduced KLF6 expression leads to increased MDM2 and reduced p53, and this imbalance in the MDM2-p53 pathway is further associated with reduced survival of HCC patients after surgical resection. Conversely, overexpression of KLF6 leads to decreased MDM2 expression and increased p53 expression in HCC cell lines [46]. Cao et al. found that the Enigma LIM domain protein is involved in signal transduction through protein kinases, which can increase MDM2 ubiquitin ligase activity and p53 degradation [47]. In addition, Enigma can be stimulated by serum response factor (SRF), which is also overexpressed in HCC, leading to further stabilization of MDM2 and degradation of p53 [43, 44].

Levels of MDM2 and p53 are significantly higher in HCC tissues compared to adjacent liver tissues, and their expression levels are related to the pathological grade and prognosis. Zhang et al. demonstrated in their study that p53 and MDM2 were overexpressed in all 181 pairs of HCC tissues compared to adjacent liver tissues [48]. Tumor cells in low pathological grade HCC patients tended to express p53 higher than in high pathological grade HCC patients. Likewise, the reduced expression of MDM2 in HCC is a predictor of better survival in patients after tumor resection [48]. These studies suggest that the expression levels of p53 and MDM2 may be useful indicators for predicting the prognosis of HCC. Currently, methods for early diagnosis of HCC are limited. Changes observed in the MDM2-p53 pathway may provide a more effective and accurate way to diagnose early-stage HCC, allowing patients to undergo surgical resection earlier and significantly improving clinical outcomes in the absence of novel targeted treatments for this pathway.

In addition to breast cancer and liver cancer mentioned above, MDM2 is closely related to the development of tumors in various solid cancers, including non-small cell lung cancer, colorectal cancer, and so on.

## Research status of MDM2 inhibitors and tumor therapy

Based on a wealth of research evidence, the development of drugs targeting MDM2 has received increasing attention, and multiple targeted drugs have been developed, some of which have already advanced to clinical trials. This makes targeting MDM2 a promising direction for cancer treatment. Currently, several MDM2-targeted therapeutic drugs that have been reported more frequently include AMG232, Chalcones, Fluspirilene, Hoiamide, and several others (Table 1).

**Table 1:** Targeted therapy drugs of MDM2, mechanism and clinical trials

Medicine	Mechanism	Clinical trial	References
AMG 232	The meta-chlorophenyl, para-chlorophenyl, and c-chain isopropyl moieties of AMG 232 interact with MDM2, inhibiting degradation, enhancing its stability, and promoting transcriptional activity	Phase 1	[51]
Chalcones	Chalcones feature an unsaturated carbonyl group positioned between two phenyl rings, which can impede the interaction between MDM2 and p53	–	[58, 59]
Fluspirilene	Its two fluorophenyl and phenyl groups can occupy either the Leu26 or Phe19 pocket. The orientation of the molecule can be changed relative to the groups in the Phe19 and Leu26 pockets	–	[62]
Hoiamide D	Entering the hydrophobic binding pocket of MDM2, blocking the interaction between p53 and MDM2	–	[63]
Indole-3-carbinol(I3C)	Inhibiting the p53-MDM2 interaction through phosphorylation at the Ser15 site	–	[65]
Isootomolide A (IKA)	Phosphorylation of the residue Ser15 on p53 prevents MDM2-mediated degradation, thereby increasing the stability and activity of p53	–	[67]
lithocholic acid(LCA)	LCA binds to MDM2 and disrupts the p53-MDM2 interaction	–	[68]
Nutlins	Inhibiting the formation of the p53/MDM2 complex by binding to MDM2, thereby enhancing the stability of the p53 protein through post-translational modifications	–	[33, 69]
Nutlin-1	The halogen on its phenyl ring is chlorine, which facilitates the substitution of residues Leu26 and Trp23 on the phenyl ring of p53, while the last residue Phe19 is replaced by its isopropoxy group	–	[27]
Nutlin-2	The halogen on its phenyl ring is bromine, facilitating the substitution of residues Leu26 and Trp23 on the phenyl ring of p53, while the last residue Phe19 is replaced by an ethoxy group	–	[27]
RG7112	Activating the p53 pathway in vivo induces apoptosis in tumor cells	Phase 1	[81, 82]
Idasanutlin (RG7388)	Inhibiting the formation of the p53/MDM2 complex by binding to MDM2, thereby increasing the stability of the p53 protein through post-translational modifications	Phase 3	[86]
SAR405838 (MI-77301)	Binding to MDM2 through a pocket similar to the MDM2 binding pocket, inhibiting the p53-MDM2 interaction	–	[88]
ALRN-6924	Mimicking the alpha-helical activation domain of p53 to bind with high affinity to MDMX and MDM2	–	[89]
NVP-CGM097	After binding to MDM2, the structure features a dihydroisoquinolinone scaffold positioned in the middle of the MDM2 active pocket. Serving as a scaffold connecting three crucial moieties, it occupies the three key active cavities of Leu26, Trp23, and Phe19	Phase 1	[91]
HDM201	Intermittent pulse high-dose treatment with HDM201 induces the expression of PUMA (p53 upregulated modulator of apoptosis) and cell apoptosis in preclinical models, achieving in vivo anti-tumor activity	–	[95]
APG-115	APG-115 enhances the anti-tumor effects of PD-1 antibodies in Trp53wt, Trp53mut, and Trp53-deficient (Trp53−/−) homologous tumor models	–	[105]

### AMG 232

AMG 232 is an orally active MDM2 inhibitor [49, 50]. AMG 232 binds to the hydrophobic binding pocket of MDM2. At Phe19, Trp23, and Leu26 residues, the m-chlorobenzene, p-chlorobenzene, and C-chain isopropyl groups of AMG 232 interact with MDM2 to inhibit MDM2 degradation and improve its stability and transcriptional activity [51]. In a Phase I trial, AMG232 demonstrated a favorable safety profile and pharmacokinetic parameters in patients with p53 WT advanced solid tumors or multiple myeloma and controlled disease progression [52]. A phase I clinical trial in AML patients showed that although AMG232 has severe gastrointestinal adverse reactions at high doses, its pharmacokinetic parameters, targeting, and clinical activity are objective. According to Sahin et al. AMG 232 can induce activation of p53 in ovarian cancer cells. In their study, OVTOKO, OVMANA, and TOV-21G cells were treated with AMG 232, and p53 and its target gene p21 were activated in all cell lines tested. However, in OVTOKO and OVMANA, cell lines with high MDM2 expression showed some resistance to treatment [49]. The SJSA-1 osteosarcoma model, showed significant anti-tumor activity, significantly promoting tumor regression without noticeable toxic side effects [25, 53]. In addition, some studies have combined AMG232 and platinum containing agents in NCH-460 non-small cell lung cancer model. Both drugs can inhibit tumor growth, and the combined treatment can also produce synergistic anti-tumor effects. At the same time, AMG232 combined with inotecan (also known as CTT-11) was used in HT116 colorectal cancer model, and the combined treatment significantly improved the anti-tumor efficacy compared with a single drug [54]. AMG232 has synergistic cytotoxic effects with MEK inhibitors and DNA damage-inducing chemotherapy drugs, and combination treatment can significantly enhance the anti-AML effect in vivo [55]. A phase I clinical trial for metastatic melanoma showed that AMG232 combined with trametinib or dabrafenib had tolerable doses, safety, and pharmacokinetic parameters, and showed early anti-tumor activity [56]. In addition, AMG232 combined with anti-PD-1 antibodies can enhance T-cell killing of tumor cells, with the potential to overcome resistance to immunotherapy [57].

### Chalcones and derivatives

Chalcones are a type of flavonoid and are natural products found in many fruits and vegetables. They have 15 carbon atoms in their chemical structure and have antibacterial, antifungal, and anti-inflammatory effects. All Chalcones have an unsaturated carbonyl group between the two phenyl rings, which can prevent the interaction between MDM2 and p53 [58, 59]. Chalcone derivatives include A, B, and C, which can inhibit the p53-MDM2 interaction by directly binding to MDM2 or denaturing MDM2. Chalcones D does not affect the p53-MDM2 interaction, so it is used as a negative control in studies involving other chalcones [60]. Derivatives A, B, and C have similar chemical structures. Compound A has a chlorine atom on the carbonyl group of one phenyl ring and an acid group at the end of the other phenyl ring. Chalcones B has an extra chlorine group on the same ring, while C has two methyl groups on the carboxylic acid functional group attached to the carbon atom alpha. Compounds A and C, each with only one chlorine group on the ring, interact with MDM2 through a single substituted phenyl, similar to tryptophan 23 (Trp23) residues. On the other hand, compound B does not directly bind to MDM2 like A and C, but denatures MDM2 [60]. This reveals the importance of functional groups, as compound A further chlorinates at the para position in addition to the substitution of chlorine. This may create steric hindrance, preventing direct binding of compound B to MDM2, and forcing it to inhibit the interaction through a different mechanism. Similar to Chalcones derivatives A, B, and C, boronic chalcones can bind to MDM2, upregulate p53, and thereby inhibit cancer cells, particularly the breast cancer cell line (MDA-MB-231). Kumar et al. found that the IC50 value for growth inhibition of MDA-MB-231 cells treated with these compounds was significantly lower than that of normal breast cell lines. The effect of boronic chalcones is dependent on p53, and due to the low level of p53 in normal breast epithelial cells, the effect of boronic chalcones is not obvious. Although boronic chalcones can successfully bind to MDM2 and prevent MDM2-induced degradation of p53, these compounds are less effective in normal cells compared to cancer cells.

### Fluspirilene

Fluspirilene is an injectable antipsychotic drug used to treat patients with chronic schizophrenia [61]. Although this compound has been used to treat schizophrenia, recent studies have shown that Fluspirilene can also play a role in inhibiting the p53-MDM2 interaction. Fluspirilene disrupts the interaction by filling the hydrophobic binding pocket and binding to the MDM2 site. The compound can bind to MDM2 in two ways, with the two fluorophenyl and phenyl groups occupying the Leu26 or Phe19, and the fluorophenyl and phenyl groups can be loaded into the Leu26 and Phe19 pockets, respectively [62]. On the other hand, the orientation of the molecule can be changed relative to the groups in the Phe19 and Leu26 pockets [62]. Therefore, Fluspirilene binds competitively to MDM2, preventing MDM2 from binding to p53. In vitro studies have found that Fluspirilene can inhibit the growth of HCT116 cells or other colon cancer-related cell lines. However, Fluspirilene can only act as an inhibitor when p53 is functional, as the percentage of cell growth in HCT116-p53 knockout (−/−) cells is significantly higher than that in HCT116-p53 wild-type (+/+) cells [62]. Therefore, Fluspirilene may be another important avenue for improving cancer treatment, as it is a p53-dependent inhibitor targeting the p53-MDM2 interaction and reducing cancer cell growth.

### Hoiamide D

Hoiamide D is a P53-MDM2 interaction inhibitor obtained from marine cyanobacteria. Hoiamide D contains leucine and phenylalanine [63], which may be an important reason for Hoiamide D as an inhibitor because p53 also has similar amino acids such as Leu26 and Phe19. Like p53, Hoiamide D enters MDM2’s hydrophobic binding pocket, blocking the interaction between p53 and MDM2. Malloy et al. conducted experiments in non-small cell lung cancer cell lines H460 or NCI-H460, finding that Hoiamide D inhibited the growth of these cells [63], and this inhibition is achieved by preventing the binding of MDM2 to p53. Therefore, Hoiamide D, as an important natural product and MDM2 inhibitor, deserves further research to explore its potential for improving lung cancer treatment.

### Indole-3-carbinol (I3C)

Indole-3-carbinol (I3C) is a compound extracted from cruciferous vegetables such as cauliflower, broccoli, and cabbage. It is a phytochemical produced by indole-3 decomposition, which also reacts with itself and other metabolites to form conjugates, including indole-3-tryptophan and indole-3-carboxaldehyde [64]. I3C inhibits the p53-MDM2 interaction by p53 phosphorylation. Since all three hydrophobic binding pockets on MDM2 (usually occupied by residues Phe19, Trp23, and Leu26) need to be filled to successfully interact with p53, and I3C's phosphorylation of p53 mainly occurs in its N-terminal region, particularly at Ser15, which prevents MDM2 from binding to p53. I3C not only inhibits the p53-MDM2 interaction by phosphorylating the Ser15 site but also induces cell cycle arrest in breast cancer cells by p53 phosphorylation. For example, when I3C is treated with the non-cancerous human breast epithelial cell line MCF10A, cell cycle arrest occurs in the G1 phase, and the effect of I3C on cell cycle arrest is mediated by p53 because cell cycle arrest was observed in MCF10A cells stably expressing dominant-negative p53 [65]. Therefore, if p53 is present, I3C can induce p53 phosphorylation, then activate p53 and ATM (Ataxia Telangiectasia Mutated), and ATM activation leads to inhibition of the p53-MDM2 interaction and G1 cell cycle arrest [65].

### Isootomolide A (IKA)

Isootomolide A (IKA) is a compound extracted from the plant cinnamon, found in the leaves of the species, which inhibits the interaction between p53 and MDM2 by p53 phosphorylation [66]. It phosphorylates p53 residue Ser15, one of the amino acids on p53 that directly interacts with MDM2 to bind. Since the introduction of this compound makes Ser15 unavailable, MDM2 cannot bind to p53 due to one of its hydrophobic pockets being unoccupied, preventing MDM2-mediated p53 degradation, thereby increasing p53 stability and activity. IKA blocks the binding of MDM2 to p53 and then inhibits the growth of lung cancer cells. For example, after IKA treatment of lung epithelial cell line A549, the proliferation of these cells is inhibited [67]. Chen et al. found that IKA has an IC50 of 4.4 µM, and complete inhibition of cell growth requires 10 µM. In addition, like other small molecules targeting the p53-MDM2 interaction, IKA promotes cell cycle arrest and apoptosis. Cell cycle arrest occurs between G0 and G1 phases and produces significant effects at 6 h [66]. Therefore, IKA is another natural product that can destroy the p53-MDM2 binding through p53 phosphorylation and induce cell cycle arrest and apoptosis.

### Lithocholic acid (LCA)

Lithocholic acid (LCA) is an endogenous steroidal bile acid that binds to MDM2 and blocks the p53-MDM2 interaction [68]. As a secondary bile acid, LCA is commonly found in bile and is used to dissolve fat for absorption. The compound consists of three cyclohexanes and one cyclopentane, which is linked to a carboxylic acid. Notably, high levels of LCA increase the risk of cancer, including colon cancer. Although high concentrations of LCA are toxic and carcinogenic, studies have shown that it can also reduce the risk of cancer development by blocking the p53-MDM2 interaction and inducing apoptosis. For example, as a dual inhibitor, LCA can bind to both MDM2 and MDM4. MDM4, also known as MDMX, regulates p53 levels similarly to MDM2 [68]. The dissociation constants of MDM4 and MDM2 with LCA are 15.4 µM and 66.0 µM, respectively, so MDM4 has a slightly stronger binding affinity for the compound than MDM2. Despite this difference, LCA can successfully bind to MDM2, thereby increasing p53 stability. However, altering and modifying the chemical structure of LCA weakens its binding affinity. In vitro studies have shown that LCA induced apoptosis in colon cancer cells, such as the human colon cancer cell line HCT116. Treatment of HCT116 cells with a concentration of 300 µM LCA increases the activation of apoptosis markers caspase 3 and 7 [68]. This apoptotic effect in HCT116 can be explained by LCA binding to MDM2 and MDM4, resulting in elevated p53 levels.

### Nutlins

Nutlins is a family of MDM2 inhibitors discovered by Vassilev et al., consisting of Nutlin-1, Nutlin-2, and Nutlin-3. They act by binding to MDM2 to inhibit the formation of the p53/MDM2 complex, thereby increasing the stability of the p53 protein through post-translational modifications [32, 69]. The core structure of Nutlins is cis-imidazoline. This structure was discovered through in vivo experiments, to find small molecule inhibitors of MDM2 through multiple chemical modifications. Nutlins with a cis-imidazoline structure were the first small molecules shown to bind to MDM2 and block its specific binding to p53 [70]. This was achieved by adding functional groups such as halogenated benzene rings, mimicking the hydrophobic p53 amino acid residues Phe19, Trp23, and Leu26. In the Nutlin family, Nutlin-3 has been shown to have a higher specificity for inhibiting the interaction of MDM2-p53 and induces p53 stabilization and activation more significantly [71, 72]. Therefore, Nutlin-3 is the most used and evaluated in cancer treatment research. Nutlin-1 contains three functional groups, which mimic certain residues of p53 when bound to MDM2 [73], mainly including two halogenated benzene rings and an isopropoxy group on the methoxybenzene ring. The halogens on the benzene rings are chlorine, which helps replace the p53 residues Leu26 and Trp23, and the last residue Phe19 is replaced by an isopropoxy group [27]. On the other hand, although Nutlin-2 retains the two halogenated benzene rings, the halogen used is bromine instead of chlorine, and the isopropoxy group replacing Phe19 is replaced by ether. Like Nutlin-1 and Nutlin-3, the brominated benzene rings also fill the binding pockets of Leu26 and Trp23, while the ether substituent is located in the binding pocket of Phe19. As an inhibitor of p53-MDM2 interaction, Nutlin-3a binds MDM2 150 times more powerfully than Nutlin-3b.

Nutlin-3 can increase the levels of p53, thereby activating its function and slowing down the DNA repair process in colon cancer cells [74]. Inducing DNA damage response in the S phase of the cell cycle, with γ-h2ax as a marker for DNA damage and repair, the number of γ-h2ax increases after treatment with Nutlin-3 [75]. However, since the number of γ-h2ax decreases after repeated use of Nutlin-3, it cannot be used alone to treat colon cancer. In the treatment of lung cancer, radiotherapy combined with Nutlin-3 enhances the cytotoxicity of ionizing radiation (IR), helping to increase cell sensitivity and induce cell death. Therefore, in combination therapy, Nutlin-3 sensitizes many types of cancer cells but has less effect in patients with low MDM2 expression [76]. Nutlin-3 stabilizes and activates p53, induces G1 and G2 phase cell cycle arrest and apoptosis in osteosarcoma (OS) cells, and is based on p53-MDM2, meaning that this apoptosis and growth inhibition occurs in OS cells with wild-type p53, such as U-2 OS cells, but not MG63 and SaOS2 cells with mutant p53 or deletion of p53 do not exhibit Nutlin-3 dependent growth inhibition [77].

P53 mutations are rare in nasopharyngeal carcinoma (NPC) [78], suggesting that the p53-MDM2 interaction may be a therapeutic target for nasopharyngeal carcinoma cells. After testing the effect of Nutlin-3 on nasopharyngeal carcinoma and nasopharyngeal epithelial (NPE) cell lines, it was found that the drug more strongly inhibited the p53-MDM2 interaction in nasopharyngeal carcinoma cells (C666-1) than in NPE cells (NP69 and NP460) [79]. Furthermore, in the treatment of nasopharyngeal carcinoma, studies have shown that Nutlin-3 has a synergistic effect with cisplatin. However, further studies have shown that long-term use of Nutlin-3 reduces sensitivity in C666-1 cells [79]. Despite its limitations, Nutlin-3 is a highly promising anticancer drug that may be used in the future to improve the efficacy of nasopharyngeal carcinoma chemotherapy.

RG7112 is an improvement on the Nutlins structure by Roche and is the first drug to enter clinical trials. RG7112 has been shown in vitro to have growth inhibitory and cytotoxic effects on MDM2 protein highly expressed SJSA-1 osteosarcoma cells. In HCT116 and SJSA1 cells G1 and G2/M phases, RG7112 induces dose-dependent cell cycle arrest [80]. In vivo experiments have shown that RG7112 (25 ~ 200 mg/kg, oral) activates the p53 pathway in the body, inducing tumor cell apoptosis. RG7112 (100 mg/kg^−1^, once a day, 5 days a week, for 3 weeks) reduced the tumor growth rate of the GBM model and increased the tumor survival rate [81]. Currently, RG7112 has been tested in a variety of clinical trials, mainly including chronic myeloid leukemia (CML), acute myeloid leukemia (AML), solid tumors, and hematological tumors. Although the use of this drug in liposarcoma patients showed a significant increase in p53 and downstream p21 levels, all patients experienced at least one adverse reaction [82]. Another phase I clinical trial in leukemia patients also showed that RG7112 treatment can increase the expression levels of p53 and downstream genes. Of the 30 patients, 5 had complete response (CR) or partial response (PR) and 9 had stable disease (SD) [83]. However, due to the poor tolerance of RG7112 and the relatively severe hematological and gastrointestinal toxicity at the required high doses, no further trials were conducted.

Idasanutlin (RG7388) is a second-generation Nutlins, designed to improve the efficacy of first-generation Nutlins [83]. With improved efficacy, Idasanutlin is the only MDM2 antagonist to enter Phase III clinical trials as of 2018 [84]. In a mouse SJSA xenograft model, RG7388 (25 mg/kg orally) inhibited tumor growth and regression, and induced apoptosis and anti-proliferation effects [85]. According to data from the China Medicinal Pipeline Monitoring Database (CPM), RG7388 has undergone 15 clinical trials, with the highest global research and development status being a Phase III clinical trial (NCT02545283) in combination with cytarabine to treat relapsed or refractory AML. Preliminary results showed a complete remission rate of 25% and a median remission time of about 6.4 months, with efficacy related to pre-treatment MDM2 protein levels [86]. However, the study was terminated due to unexpected results. RO6839921 is a non-active polyethylene glycolated prodrug of RG7388, designed to improve the exposure variability and pharmacokinetic characteristics of RG7388. Safe dose intravenous injection of RO6839921 showed good anti-tumor activity in osteosarcoma and AML xenograft models [87]. Although Phase I studies showed that RG7388 improved the pharmacokinetic parameters of advanced solid tumors and AML, its safety was comparable to RG7388, showing no significant advantages.

### SAR405838 (MI-77301)

SAR405838 (MI-77301) is a new drug that inhibits the p53-MDM2 interaction by binding to MDM2. Like other compounds that bind to MDM2, SAR405838 has three binding pockets similar to MDM2. Therefore, SAR405838 can compete with p53 and bind to MDM2. SAR405838 not only utilizes the binding pocket but also interacts with MDM2 using the Cl atom [88]. Several contact areas between SAR405838 and MDM2 form the SAR405838-MDM2 complex, which then activates p53 to exert its tumor suppressor function. However, the drug treatment also has some limitations, such as dependence on p53. SAR405838 inhibits the growth of cancer cells and increases p53 levels by binding to MDM2, but when p53 is mutated or deleted, the efficacy of the drug is reduced [88]. Despite some limitations, SAR405838 is still a potential compound for the treatment of a variety of cancers, including colorectal cancer and prostate cancer, due to its ability to inhibit the growth of cancer cells. Wang et al. found that SAR405838 inhibits the growth of cancer cells [88]. In their experiment, the authors orally administered 200 mg/kg of SAR405838 to HCT-116 cell-bearing mice daily for about 3 weeks. The results showed that SAR405838 was able to inhibit the growth of mouse colon cancer xenografts [88]. SAR405838 was also able to inhibit the growth of prostate cancer cells, especially LNCaP (lymph node prostate cancer) cells. Wang et al. orally administered 100 mg/kg of SAR405838 to mice daily for 4 weeks, and the results showed that the drug induced growth inhibition of prostate cancer cells. In addition, the authors demonstrated that a dose of 200 mg/kg could induce 80% regression [88]. Although twice the amount of drug was used to inhibit growth, the experiment showed no toxicity to mice.

### ALRN-6924

ALRN-6924 is a stable, cell-penetrating α-helical peptide that mimics the a-helical activation domain of p53 and binds to MDMX and MDM2 with high affinity, making it a dual MDM2/MDMX inhibitor. ALRN-6924 represents a new class of intracellular protein–protein interaction inhibitors, showing antitumor activity in TP53 wild-type hematological malignancies and solid tumor mouse models, providing a theoretical basis for clinical translation [89]. Mansoor N. Saleh et al. evaluated the safety, pharmacokinetics, pharmacodynamics, and antitumor effects of ALRN-6924 in patients with solid tumors or lymphomas as shown in a Phase I trial, which showed: ALRN-6924 has good tolerability and antitumor activity, including durable complete and partial responses and disease control was achieved in more than half of evaluable patients [90]. Notably, the hematopoietic toxicity commonly observed with selective MDM2 inhibitors was almost absent in ALRN-6924, suggesting that dual MDM2/MDMX inhibition may allow more complete p53 activation, and its toxicity profile will allow for combination therapy and use as a chemoprotective agent. In summary, the study demonstrated the safety of the pioneering dual MDM2/MDMX inhibitor ALRN-6924. ALRN-6924 produced disease control and objective responses in various TP53-WT tumor patients, and its low hematopoietic toxicity provides a basis for clinical trials of ALRN-6924 as a chemoprotective drug for TP53 mutant cancer patients.

### NVP-CGM097

After NVP-CGM097 binds to MDM2, the dihydroisoquinolinone scaffold in its structure is located in the middle of the MDM2 active pocket, serving as a scaffold that connects three key groups and occupies the three key active cavities of Leu26, Trp23, and Phe19. The Leu-26 pockets are filled with isopropoxy and methyl ether. The ether oxygen forms water-mediated hydrogen bond interactions with the hydroxyl of tyr1-100 and the carbonyl of Gln- 24. The carbonyl in the scaffold also participates in the formation of hydrogen bonds with the carbonyl of ph-55, which contributes to the overall affinity of the molecule. More importantly, it induces conformational constraints on its adjacent n-aryl side chain, giving it the correct torsion angle to optimally enter the Phe19 active cavity. The n-methyl quinazoline binds in the water-rich region at the entrance to the Phe19 cavity, perfectly positioned between the protein walls, significantly enhancing its affinity for the MDM2 protein [91]. NVP-CGM097 and its derivative inhibitors show strong antitumor activity in patients with B-cell acute lymphocytic leukemia [91]. In combination with RAF kinase inhibitors, it synergistically inhibits Braf (v-raf murine sarcoma viral oncogene homolog B1) mutated melanoma cell proliferation [92], and in combination with anaplastic lymphoma kinase (ALK) inhibitors, it synergistically inhibits ALK mutated neuroblastoma and prolongs survival. The p53 mutant BON1 and NCI-H727 cells are resistant to NVP-CGM097, but when combined with 5-fluorouracil, it exerts a potential therapeutic effect on neuroendocrine tumors by increasing the expression of p53 and p21 [93]. A Phase I clinical trial showed that NVP-CGM097 controls the progression of solid tumors by activating the p53 pathway with acceptable pharmacokinetic parameters and safety profile.

### HDM201

HDM201 is designed by Novartis. HDM201 selectively induces cell cycle arrest and apoptosis in p53 wild-type tumor cells, with dose-dependent PK/PD parameters that inhibit the growth of SJSA-1 osteosarcoma model cells [94]. Compared to NVP-CGM097, intermittent high-dose pulse treatment with HDM201 induces clinical model PUMA (p53 upregulated modulator of apoptosis) expression and cell apoptosis, achieving in vivo antitumor activity [95]. The PKPD model of HDM201 also indicates that the antitumor activity of HDM201 is not dependent on the schedule, but is related to the cumulative dose, suggesting the feasibility of clinical intermittent administration to reduce the common hematological toxicity of MDM2 inhibitors. HDM201 in combination with Fms-like tyrosine kinase 3 (FLT3) inhibitors specifically induces apoptosis and death of FLT3-itd positive TP53 wild-type AML cells [96], suggesting that HDM201 has the potential to be combined with other targeted drugs. Phase I clinical results show that HDM201 has good pharmacokinetic parameters and safety, with safety not significantly different between tumor types and treatment regimens, but thrombocytopenia is common. Clinical studies of single-dose administration are ongoing, and HDM201 shows good anti-leukemia activity in wild-type p53 patients [97]. Phase Ib trials in AML patients show that AML patients have good tolerance and treatment response to HDM201 in combination with Bcl-2 inhibitors [98]. The safety and clinical efficacy of HDM201 in combination with LEE011 (a CDK4/6 inhibitor) for the treatment of liposarcoma has also been confirmed [99]. In patients with relapsed AML after allogeneic hematopoietic stem cell transplantation, HDM201 also has good tolerance and clinical activity [100].

### MDM2 and immunotherapy

**Fig. 1:**
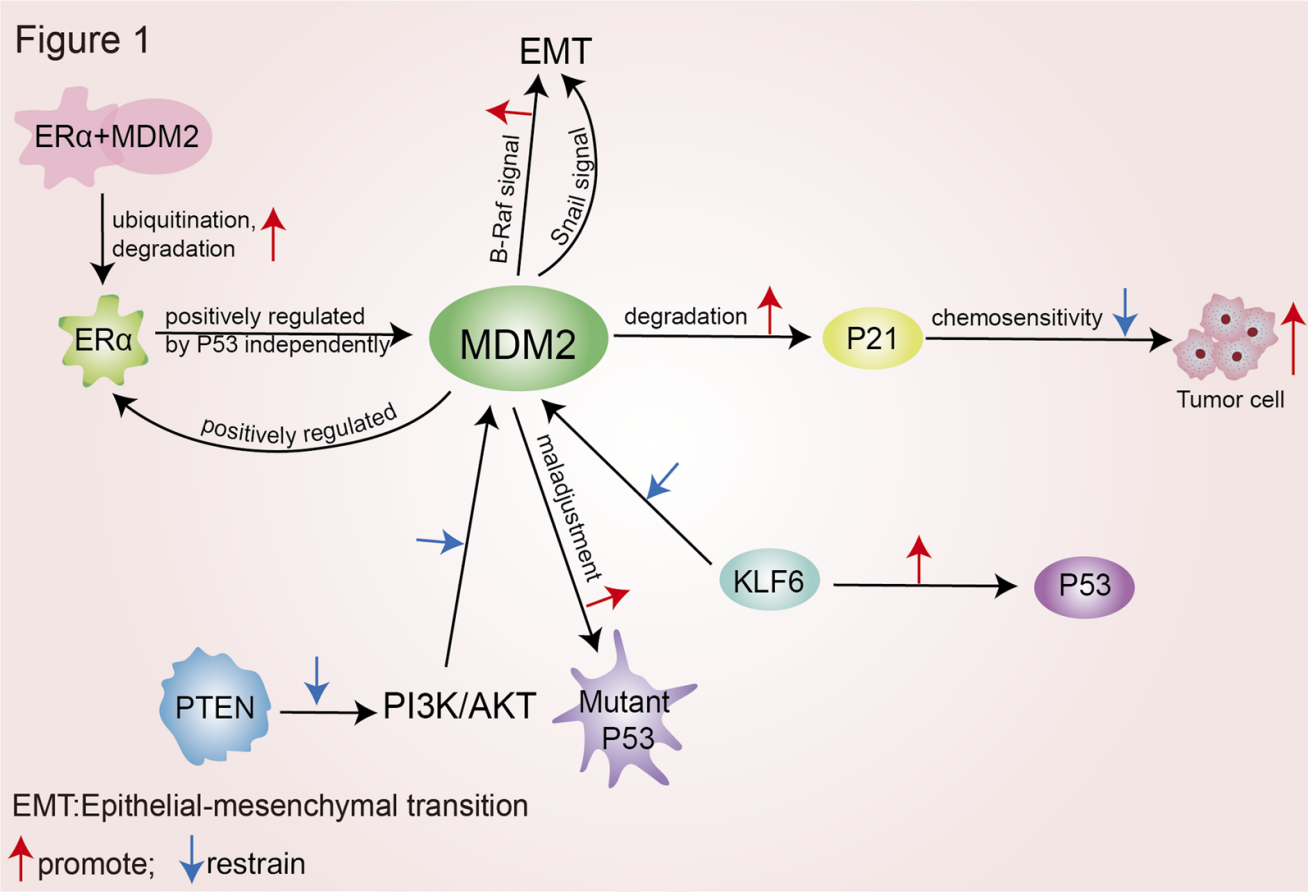
The correlation between MDM2 and tumorigenesis

Immunotherapy based on immune checkpoint inhibitors (ICIs) is a cancer treatment method that has received much attention and achieved significant success in recent years. The efficacy of immunotherapy is influenced by various factors, especially the expression and activation of some molecules, which significantly affect the efficacy of ICIs treatment. Recent reports indicate that a small percentage of patients (7–29%) may develop hyperprogressive disease (HPD), an unexpected and more serious phenomenon observed in ICIs-based immunotherapy, and its characteristic is that patients not only cannot benefit from ICIs, but also tumor growth accelerates after immunotherapy [101]. At present, although the potential resistance mechanisms of cancer immunotherapy and HPD are not yet clear, studies have shown that MDM2 over expression is highly correlated with poor prognosis and may even lead to the development of HPD (PMID: 33334611; PMID: 27827313; PMID: 30193240; PMID: 28351930). One of the studies suggests that 155 stage IV tumor patients who received ICIs treatment were evaluated, and found that time to treatment failure (TTF) < 2 months was observed in six patients with MDM2/MDM4 amplification, and four of them showed significant increases in existing tumor size (55–258%), new large masses, and significantly accelerated progression space (PMID: 28351930). Although the types of cancer reported in the literature (including bladder cancer, triple negative breast cancer, endometrial stromal sarcoma, lung cancer), types of ICIs (anti-PD1, anti-PD-L1 and even anti-CTLA-4), and treatment (first-line or non first-line ICIs treatment) were different, it was confirmed that patients with MDM2/MDM4 amplification had no positive reaction to ICIs [102–104]. Therefore, targeting MDM2/MDM4 has the potential to improve the efficacy and response rate of immunotherapy. Douglas D and others used mouse cells and tumor models to study whether APG-115 targeting the MDM2-p53 pathway could regulate the immune response and enhance the antitumor immunity induced by anti-PD-1 treatment [105]. The results showed that APG-115 activates p53 in tumor microenvironment (TME) immune cells to promote antitumor immunity, and APG-115 enhances the antitumor effects of anti-PD-1 antibodies in Trp53wt, Trp53mut, and Trp53-deficient (Trp53−/−) syngeneic tumor models. Mechanistically, in addition to the increased infiltration of cytotoxic CD8 + T cells and M1 macrophages in Trp53wt tumor TME, the reduction in M2 macrophage infiltration also helps Trp53wt and Trp53mut tumors TME transition from immunosuppressive to immunostimulatory. In Trp53 knockout mice with complete deletion of endogenous Trp53 gene, APG-115 treatment did not enhance the efficacy of anti-PD-1, indicating that a complete p53 is required to activate p53 protein in host animal immune cells. In summary, the study showed that the use of MDM2 antagonists such as APG-115 can enhance the antitumor efficacy of PD-1, importantly, this effect is independent of the p53 status of the tumor itself (Fig. 1).

## Conclusion

The binding and interaction of MDM2 with p53 play a crucial role in promoting the development of tumors. Due to the inaccessibility of drugs specifically targeting p53, targeting MDM2 and the interaction between MDM2 and p53 have become promising drug targets for cancer treatment. Disrupting this interaction is through p53 acting as a tumor suppressor to promote cell cycle arrest and apoptosis. To increase p53 levels and thus induce cancer cell death, researchers have also developed compounds that prevent MDM2 from binding and interacting with p53. Although there has been extensive research in this field to develop inhibitors of the p53-MDM2 interaction, there are still limited drugs that have entered clinical trials. There is an urgent need to develop small molecules that can inhibit the p53-MDM2 interaction and potent therapeutic agents with low or non-clinical toxicity. Although most compounds fail to enter clinical trials for various reasons, many of these compounds can serve as key lead compounds for developing more refined and effective p53-MDM2 inhibitors. The design and development of new mechanism anti-cancer drugs targeting MDM2-p53 is one of the hotspots in the global field of cancer drug research and development. However, currently only a few candidate drugs are in the clinical trial stage, and there are no marketed drugs targeting MDM2. More evidence is needed to confirm the basic mechanisms and clinical application value of these drugs.

## Data Availability

The results/data/figures in this manuscript have not been published elsewhere, nor are they under consideration (from you or one of your Contributing Authors) by another publisher.
